# Effect of inflammation on neurovascular coupling, microperfusion, and clinical outcomes in ischemic stroke patients: a case series report

**DOI:** 10.3389/fmed.2025.1665396

**Published:** 2025-10-27

**Authors:** Astrid Cancino, Pablo Muñoz, Pablo Cox, Lilian Acevedo, Sebastián Castillo, Aldo Letelier, Alejandro Veloz, Maria Rodriguez-Fernandez, Steren Chabert

**Affiliations:** ^1^PhD Program in Sciences and Engineering for Health, Universidad de Valparaíso, Valparaíso, Chile; ^2^Millenium Institute for Intelligent Healthcare Engineering iHealth, Santiago, Chile; ^3^Center of Interdisciplinary Biomedical and Engineering Research for Health – MEDING, Universidad de Valparaíso, Valparaiso, Chile; ^4^Center for Translational Research in Neuropharmacology (CITNE), Faculty of Medicine, Universidad de Valparaíso, Valparaíso, Chile; ^5^Unidad de Imagen Compleja, Hospital Carlos Van Buren, Valparaíso, Chile; ^6^Department of Neurology, Hospital Carlos Van Buren, Valparaíso, Chile; ^7^School of Biomedical Engineering, Universidad de Valparaíso, Valparaíso, Chile; ^8^Institute for Biological and Medical Engineering, Schools of Engineering, Medicine and Biological Sciences, Pontificia Universidad Católica de Chile, Santiago, Chile

**Keywords:** stroke, neuroimaging, inflammation, hemodynamics, microperfusion, outcome

## Abstract

**Introduction:**

Ischemic stroke leads to a range of sequelae that affect daily functioning. In many cases, such as wake-up strokes or late hospital arrivals, the therapeutic window for reperfusion is missed for the patient, and functional outcomes remain uncertain. The inflammatory response to ischemia plays a pivotal role in the initiation, progression, and recovery phase of stroke. Yet, a gap remains in understanding its impact on neuroimaging and clinical outcomes. This prospective case series investigates the relationship between inflammation, neuroimaging findings in the first 48 h after stroke onset, and 6-months clinical outcomes.

**Methods:**

Biomarkers of inflammation, such as C-reactive protein (*CRP*) and Interleukin 6 (*IL-6*), as well as oxidative stress (OS), were measured. Additionally, advanced neuroimaging techniques were used to assess neurovascular coupling, cerebrovascular reactivity, and intravoxel incoherent motion (IVIM) for microperfusion. After 6 months, outcomes were evaluated using the modified Rankin Scale (*mRS*), and participants were categorized into two groups: those with good outcomes (*mRS* 1–3) and those with poor outcomes (*mRS* 4–6).

**Results:**

A total of 23 wake-up stroke patients not eligible for reperfusion therapy were included: 11 with cortical ischemic lesions and 12 with subcortical or deep ischemic lesions, involving the thalamus, basal ganglia, brainstem, or cerebellum. Significant differences were observed in pseudodiffusion (D*) and delayed neurovascular coupling between patients with normal and elevated inflammatory markers. *CRP* levels showed a positive correlation with these imaging findings. Additionally, when stratified by 6-months outcomes, patients with poor recovery had higher *CRP* levels and altered contralateral cerebrovascular reactivity within the first 48 h of admission.

**Discussion:**

These preliminary findings suggest that combining inflammatory and neuroimaging markers across cortical and subcortical stroke subtypes could enhance understanding of inflammation’s role in early hemodynamic responses and long-term effects outcomes. Further research is needed to explore the broader implications of these case series representations.

## 1 Introduction

Ischemic Stroke (IS) is characterized by reduced blood and oxygen supply to specific brain regions (1), representing a growing health concern that increasingly affects younger individuals due to comorbidities and sedentary lifestyles (2). IS remains a leading cause of global mortality and disability (3), with diverse manifestations driven by mechanisms such as excitotoxicity, neuroinflammation, oxidative stress (OS), and cell death signaling (4). Acute IS necessitates rapid diagnosis and personalized treatment strategies. In recent years, a multidisciplinary approach integrating biomedical engineering, clinical neurology, cellular responses, and advanced neuroimaging has significantly enhanced our understanding of stroke pathophysiology.

Conventional IS outcome predictions rely on clinical severity scales, such as the National Institute of Health Stroke Scale (NIHSS) or ischemic lesion volume (5). Assessing the impact of the ischemic injury in the clinical context and its damage mechanisms is challenging due to various factors such as location of the injury, comorbidities, uncertain previous and vascular response, and patient heterogeneity, resulting in limited information in the acute stage of IS. Early infarct volume correlates only moderately with functional outcomes and varies nonlinearly with infarct size. Larger infarct volumes are generally associated with poorer functional status, a relationship particularly evident in moderate-sized infarcts (15–200 mL). In contrast, small infarct volumes (<15 mL) often fail to reliably predict outcomes (6). Notably, most studies have focused on middle cerebral artery strokes involving cortical infarcts, while the impact of infarct location in subcortical regions remains underexplored and challenging (7, 8).

In IS, the activation of microglia, along with monocytes and neutrophils, initiates a neuroinflammatory cascade that plays a central role in the pathophysiology of brain injury. Activated microglia release interleukin-6 (IL-6), a key proinflammatory cytokine that stimulates the liver to produce C-reactive protein (CRP), an established marker of systemic inflammation (5). This inflammatory response contributes to OS and leads to disruption of the blood-brain barrier (BBB), thereby increasing the risk of cerebral edema and exacerbating neural damage (4, 9). Isoprostanes, as products of lipid peroxidation, are recognized as sensitive markers of OS in this context (10). Together, IL-6 (11–13), CRP (14, 15), and isoprostanes (10, 16) rise progressively after stroke, offering important insights into the extent of tissue injury and its relationship with functional outcomes. However, despite their relevance, these measures are not yet routinely used in clinical practice to guide prognosis.

Advanced imaging modalities, including diffusion-weighted imaging, perfusion imaging, CT angiography, and MR angiography, are central in identifying infarct core, salvageable tissue, and vascular status in real time. Beyond these, diffusion and functional MRI techniques have been developed to probe brain microstructure, microvascular perfusion, and hemodynamic response applied in various neurological and systemic conditions. Le Bihan’s bi-exponential model for IVIM imaging enables the simultaneous assessment of tissue microstructure and microvascular perfusion by separating diffusion coefficients based on the incoherent movement of water molecules. Specifically, it distinguishes the slow diffusion component (true diffusion; *D*), reflecting tissue microstructure, from the fast pseudo-diffusion component (D*), which represents the motion of blood within capillary networks. (17–21), previous IVIM work has shown decreased parameters in acute stroke conditions (22). The functional MRI, used in stroke, in acute and chronic status, shows alteration in resting state connectivity and functional connectivity that lasts even a year (23, 24), description of hemodynamic lag in a qualitative way in acute stroke (24) and qualitative neurovascular decoupling in chronic patients (25). The use of cerebrovascular reactivity (CVR), a metric that measures how vascular tone responds to environmental demands, in vascular pathologies reveals high variability in CVR responses, even in patients with significant stenosis, and is primarily employed in chronic conditions (26). However, there is still a gap in understanding CVR during the acute phase and in providing a quantitative description of the hemodynamic response function (HRF) in acute stroke patients.

Understanding the underlying mechanisms of IS and their subsequent outcomes is challenging, and previous work has primarily focused on isolated metrics. Recognizing that a complex phenomenon cannot be fully understood through a single perspective encourages us to shift and expand the paradigm. However, to date, no studies have linked advanced hemodynamic neuroimaging to the impact of inflammation on IS during the first 48 h and its related outcomes at 6 months. An integrated approach could help us select patients for treatment more effectively, even within extended treatment windows, and better predict outcomes. This case report aims to assess the influence of inflammation on neuroimaging parameters within the first 48 h post-stroke, divided into cortical and subcortical regions, and its correlation with 6-months outcomes. The hypothesis is that increased inflammation resulting from ischemia will alter hemodynamic metrics and be related to poorer outcomes.

## 2 Materials and methods

A prospective case series was conducted between 2022 and 2023. Patients were categorized into two groups based on lesion topography: those with cortical ischemic lesions and those with subcortical ischemic lesions (including thalamus, basal ganglia, brainstem, and cerebellum). Inclusion criteria included individuals aged 18 years or older with an NIHSS score of 1 or higher who presented with ischemia affecting the anterior or posterior circulation within the first 48 h of stroke symptom onset. Exclusion criteria included eligibility for thrombolysis or thrombectomy, as these are time-sensitive interventions, and additional MRI acquisition could interfere with standard clinical workflows, presence of imminent life-threatening conditions, known autoimmune or inflammatory disorders, or active infections. Informed consent was obtained directly from participants or, when necessary, through legally authorized representatives. The study was conducted in accordance with institutional and national regulations, received ethical approval from the Regional Ethics Committee under Resolution No. 3730-2021, and complied with the Declaration of Helsinki (2013 revision).

### 2.1 Clinical measurements

Upon admission, a neurologist conducted a NIHSS evaluation, and the initial ischemic volume was calculated on the DWI using ITKSNAP^1^. Outcomes were measured at 6 months with a modified Rankin Scale (mRS), Barthel Index (BI), and Functional Independence Measure (FIM) (27–30). Based on the mRS scores, the full sample was dichotomized into good outcomes (mRS 0–3) and poor outcomes (mRS 4–6) (29, 30) for retrospective data analysis.

### 2.2 Biochemical measures

A single quantitative determination of *IL-6* was conducted using the electrochemiluminescence method in the Cobas e601 (Rocha diagnostic, Indianapolis, IN, USA) with a venous blood sample collected at admission. *CRP* was quantified using turbidimetry. Isoprostane levels for the *OS* approach were measured using an ELISA immunocompetitive assay on a microplate pre-coated with an antibody for 8-Isoprostanes, following the manufacturer’s instructions (ABCAM ab175819^®^, Cambridge, United Kingdom). Absorbance was measured at 490 nm, and isoprostane values were interpolated from a calibration curve. *CRP* was measured over three consecutive days, taking the highest value as the peak. Standard reference values are <5.18 pg/ml for *IL-6* (31) and <5.0 mg/l for *CRP* (14), used to categorize inflammation levels as low or high.

Images were acquired using an eight-channel head coil on a 1.5T Signa HDxt scanner (General Electric, Milwaukee, WI, USA). In addition to the standard stroke protocol, two sequences were included: diffusion-weighted imaging (DWI) with 16 *b*-values for IVIM assessment, and functional MRI (fMRI). Anatomical images were obtained with a 3D T1-weighted sequence (resolution 0.46 × 0.46 × 1.2 mm^3^; TR = 6 s; TE = 1.8 s; field of view = 256 mm; acquisition time = 3 min 32 s). For fMRI, a T2* weighted echo-planar imaging (EPI) sequence was used (TR = 1.75 s; TE = 60 ms; resolution 1.9 × 1.9 × 5 mm^3^). The stimulus consisted of passive bilateral wrist movements (flexion-extension at 1 Hz), cued visually. Each movement lasted 3 s, followed by interstimulus intervals of 15–30 s, totaling 11 activations over 5 min. Different trained operators conducted the stimulation sessions using standardized cues. DWI was acquired using a spin-echo EPI sequence (resolution 3 × 3 × 5 mm^3^; TE = 96.6 ms; TR = 3 s; 20 slices) with the following *b*-values: 0, 40, 50, 60, 150, 160, 170, 190, 200, 260, 440, 560, 600, 700, 980, and 1000 s/mm^2^ (32). DWI acquisition time was 4 min 42 s.

### 2.3 Image analysis

The regions of interest (ROIs) for the MRI techniques were manually segmented using ITKSNAP (Version 4.01) (33). The ROIs were categorized based on the indirect and direct impacts of ischemia. Furthermore, the ischemic side is indicated by (*i*), while (*c*) denotes the evaluated contralateral region, as detailed below.

### 2.4 Nonfocal ischemic lesion impact

In the fMRI images, ROIs were drawn in the postcentral gyrus (PCG), identified in both hemispheres using the Allen Brain Atlas. The coordinates for the right PCG were (56, −11, 43), and for the left PCG, they were (−61, −13, 43). A sphere with a 10 mm radius was centered on these coordinates, with a 5 mm sphere at the peak voxel activation to account for participant variations.

Functional MRI preprocessing was conducted using SPM 12 software at the London Wellcome Trust Center for Neuroimaging. This included slice timing correction, image realignment, co-registration of functional and anatomical images, and applying 6 mm FWHM smoothing. Volumes with excessive movement were excluded based on voxel size (2 × 2 × 2 mm), with a maximum tolerance of one voxel (34). The first five volumes were removed to ensure stable tissue magnetization signals.

Activation maps were generated using a general linear model (GLM) combined with a finite impulse response (FIR) approach, as previously applied in a stroke study (25), implemented in SPM12. BOLD signal extraction was performed exclusively on validated and activated clusters, ensuring that only robust components were included in the analysis. The extraction was limited to the bilateral postcentral gyrus (PCG) ROIs to characterize the HRF curve using four parameters: Height, Time to Peak (TTP), Full Width at Half Maximum (Width), and Onset (O). HRF estimation was performed by fitting the BOLD signal to a double-gamma function using least-squares minimization in MATLAB, as implemented in the sHRF toolkit (35). The model was initialized with V0 = [6 7 1 1 16 1 0], lower bounds = [0 2 0.5 0 6 0 −20], and upper bounds = [15 10 2 10 25 1.5 20]. Fits were visually inspected, and all residuals followed a normal distribution. HRF parameters were then used to evaluate neurovascular coupling in the ischemic and contralateral hemispheres

### 2.5 Focal ischemic lesion impact

In the IVIM and CVR images, ROIs were drawn directly within the ischemic core using DWI in the subject’s native space, with the contralateral region defined as the homologous counterpart. An experienced neuroradiologist (PC) reviewed and corrected all ROIs.

DWI image analysis involved preprocessing to correct motion and Eddy current artifacts with DSI software^2^. ROIs were adjusted to reduce CSF contamination (36) by manually removing voxels near the sulci or ventricles using ITKSNAP.


(1) $$S\left(b\right)\;=\;S0\;\left[fe^{-bD^{*}}+\left(1-f\right)\;e^{-bD}\right]$$

Equation 1, where S is the signal magnitude of the DWI, and S0 is the signal magnitude without diffusion weighting. It was used to estimate the perfusion fraction (*f*), diffusion (*D*), and pseudo-diffusion (D*) using a two-step adjustment method that considered the 16 *b*-values (32, 37). First, *b*-values over 200 s/mm^2^ adjusted *D* to a monoexponential decay. Then, a bi-exponential function in MATLAB (MathWorks, Natick, MA, USA), employing the trust-region reflective algorithm, adjusting *f* and D* across all *b*-values, with boundaries of (0 4e^3^) for the first step, and (0 1) for *f* in the second step.

The CVR maps were obtained using the amplitude of low-frequency fluctuations (ALFF) in the BOLD signal from resting-state fMRI (38). The signal associated with wrist movement was removed, and the residual BOLD fluctuations represent the resting-state signal, as described before by (39, 40). From this resting-state signal, the ALFF at [0.01–0.09 Hz] was computed: the square root of the power spectrum is averaged across the low-frequency band for each voxel to produce this pixel ALFF value (41). Each pixel value is then normalized relative to the subject’s brain average. The maps obtained in this way represent cerebrovascular reactivity, and this approach has previously been used in both healthy populations and Moyamoya patients (42). ALFF maps were generated using the CONN toolbox version 22, following a standard preprocessing pipeline. Once the maps were computed, CVR values were extracted from the ischemic ROI (*CVR*_*i*_) and the contralateral ROI (*CVR*_*c*_). The difference between contralateral and ischemic ROIs was calculated for each subject, ΔCVR (CVR_*c*_ − CVR_*i*_). Positive values reflect greater reactivity in the contralateral ROI, negative values indicate greater reactivity in the ischemic ROI, and values close to zero indicate maintained reactivity between hemispheres.

Statistical analysis included descriptive statistics for all variables, such as means, standard deviations, medians, and ranges. The Shapiro-Wilk test evaluated normality. The Mann-Whitney test assessed differences in ischemic location, inflammatory levels, and outcomes in clinical measures and neuroimaging parameters (HRF, IVIM, and CVR), as well as between ischemic regions and contralateral ROIs in neuroimaging parameters. In all figures, the ischemic side is represented as the ipsilateral hemisphere, and the opposite side as the contralateral hemisphere. Pearson correlation analysis examined relationships among inflammatory markers, oxidative stress metrics, neuroimaging parameters, and clinical outcomes. Statistical significance was set at 95% confidence (alpha = 0.05). Analyses were performed using Jamovi (version 2.5), an open-source software from the Jamovi Project (2024).

## 3 Results

A total of 23 patients with ischemic stroke within the first 48 h were analyzed, including five females. The mean hospital arrival time for participants was 19.1 ± 12.4 h. All patients received standardized post-stroke care according to routine clinical protocols. Regarding ischemic location, eleven cases involved cortical regions, and twelve involved subcortical regions. The distribution of comorbidities was generally balanced between cortical (*n* = 8) and subcortical (*n* = 9) cases regarding hypertension. Diabetes mellitus was more common in patients with subcortical lesions (9 vs. 5 cortical), while cardiac illness was more frequent in cortical cases (6 vs. 2 subcortical). Smoking habits showed no relevant difference between groups (6 cortical vs. 5 subcortical), and stroke recurrence was slightly more frequent in cortical cases (5 vs. 3 subcortical). The remaining patients in each category did not present the respective comorbidity. Patients with cortical lesions showed larger lesion volumes (22.2 ± 38.5 ml vs. 4.39 ± 10.1 ml) and higher initial clinical severity as measured by the NIHSS (7.73 ± 7.43 vs. 3.75 ± 1.71) compared to the subcortical group. Inflammatory markers were also elevated in the cortical group, with higher *IL-6* (17.3 ± 11.8 pg/ml vs. 11.4 ± 13.2 pg/ml) and *CRP* levels (24.4 ± 23.8 mg/L vs. 9.88 ± 12.8 mg/L). This pattern extended to *OS*, as reflected by higher isoprostanes levels in the cortical subgroup (683 ± 122 pg/ml vs. 582 ± 86.3 pg/ml). Only volume (*p* = 0.004) and *CRP* level (*p* = 0.031) showed statistically significant differences between cortical and subcortical strokes, suggesting a stronger inflammatory response and a tendency of higher oxidative component associated with cortical strokes. Table 1 summarizes the observations of the case reports related to clinical and biochemical measures. The clinical assessments at 6 months report that 9 cases had poor outcomes, while 13 cases had good outcomes. One participant could not be contacted.

**TABLE 1:** Clinical and biochemical data by ischemic location.

Location	*N*	Age (years)	Volume (ml)	NIHSS	IL-6 (pg/ml)	CRP (mg/L)	Isoprostane (pg/ml)
Cortical	11	70.2 ± 12.6 (49–88)	22.2 ± 38.5 (0.97–122)	7.73 ± 7.43 (1–21)	17.3 ± 11.8 (1.99–40.2)	24.4 ± 23.8 (0.8–65.6)	683 ± 122 (537–901)
Subcortical	12	63.4 ± 12.2 (44–78)	4.39 ± 10.1 (0.8–34.4)	3.75 ± 1.71 (1–7)	11.4 ± 13.2 (1.5–39.2)	9.88 ± 12.8 (1.6–43.2)	582 ± 86.3 (466–731)
UMW *p*-value		0.218	0.004**	0.4	0.156	0.031*	0.051

*p < 0.05;

**p < 0.01. NIHSS, severity stroke score; IL-6, interleucin-6; CRP, C reactive protein.

**FIGURE 1:**
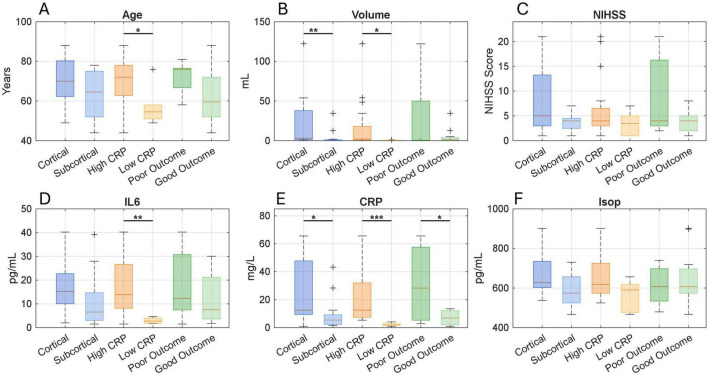
Comparison of demographic and biochemical markers stratified by lesion location (cortical vs. subcortical), systemic inflammation level (high CRP vs. low CRP), and functional outcome (poor vs. good). Boxplots display the distribution of **(A)** age, **(B)** infarct volume, **(C)** NIH Stroke Scale (NIHSS) score at admission, **(D)** interleukin-6 (IL-6), **(E)** C-reactive protein (CRP), and **(F)** isoprostane levels. Each boxplot shows the median and interquartile range, while + symbols indicate outliers. Statistical comparisons were performed using the Mann–Whitney test. Asterisks indicate significance levels: *p < 0.05, **p < 0.01, ***p < 0.001. Clinical and biochemical measures across inflammation levels, lesion location, and outcome.

Clinical and biochemical profiles (age, lesion volume, NIHSS, *IL-6*, *CRP*, and isoprostanes) are presented across subgroups categorized by lesion location (cortical vs. subcortical), inflammation level, and clinical outcome in Figure 1. Patients with high inflammation tend to be older, have larger lesion volumes, and present with higher NIHSS scores. Age differences between inflammation levels reach statistical significance (*p* = 0.035), as does volume (*p* = 0.039). No significant difference was observed in NIHSS scores across different inflammation levels. A trend toward higher isoprostane levels was noted in cases with cortical damage and elevated inflammation. These patterns emphasize the interaction between inflammatory response, OS, and clinical severity, especially in cortical injuries.

**FIGURE 2:**
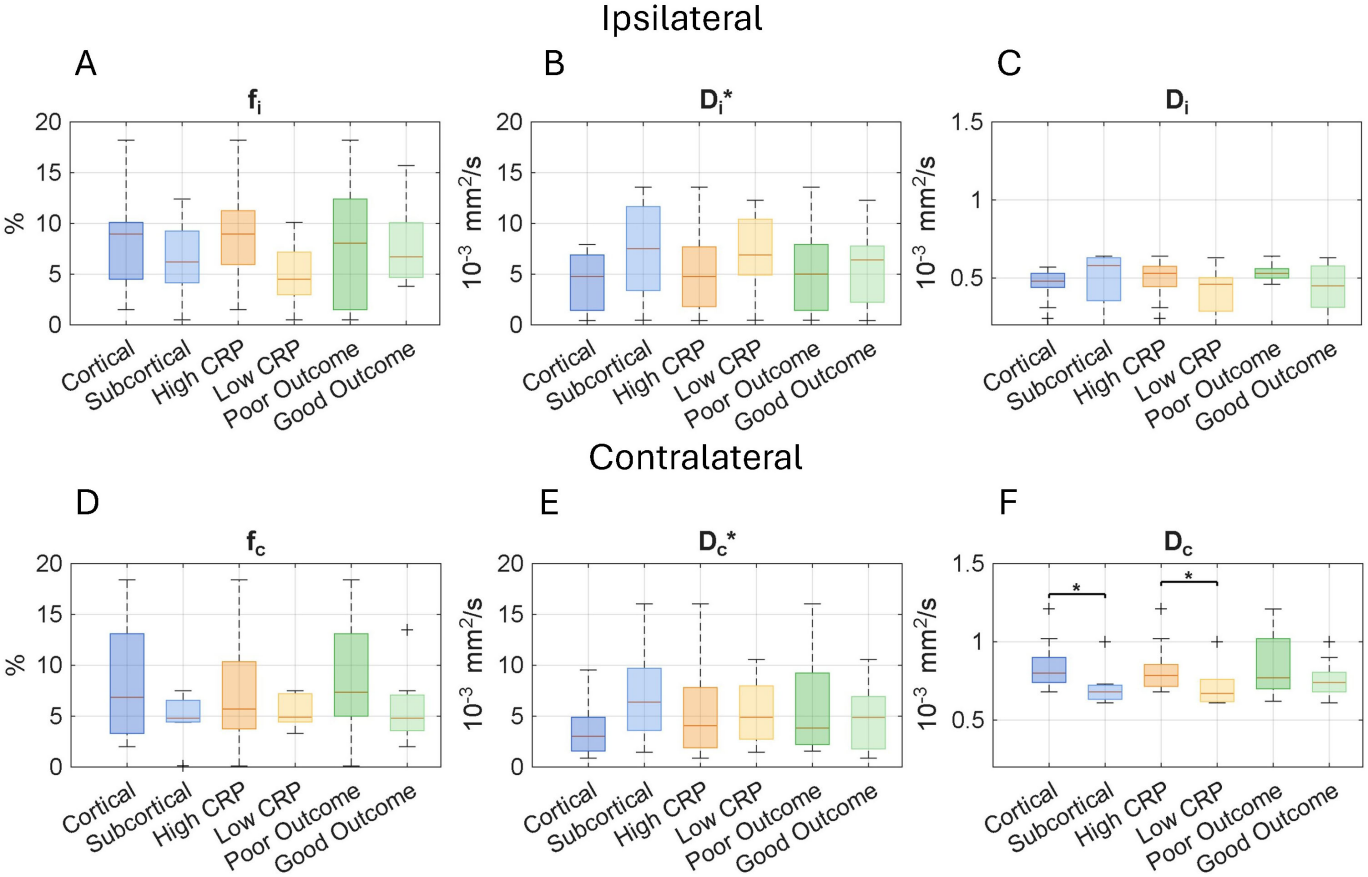
Neuroimaging parameters derived from the intravoxel incoherent motion (IVIM) model across lesion location (cortical vs. non-cortical), systemic inflammation level (high vs. low), and functional outcome (good vs. poor). Boxplots display values for the ipsilateral hemisphere (top row) and the contralateral hemisphere (bottom row); **(A,D)** Perfusion fraction (f), **(B,E)** Pseudodiffusion coefficient (D), and **(C,F)** Diffusion coefficient (D). Each boxplot shows the median and interquartile range, while **+** symbols indicate outliers. The perfusion-related parameters (f and D*) reflect microvascular perfusion, while D represents true molecular diffusion. Comparisons were conducted using the Mann–Whitney U test. Asterisks indicate statistical significance: *p < 0.05. IVIM-based microperfusion and diffusion across lesion location, inflammation, and outcome.

Focal microperfusion and microstructural analyses in Figure 2 revealed that only the *D*_*c*_ had a statistically significant difference between cortical and subcortical regions (*p* = 0.028). Across both hemispheres, D* values tended to be lower in cortical regions and higher in subcortical areas. These regional differences were notable when compared with healthy cortical reference values (*f*: 13.19% ± 2.4%; D*: 15.56 ± 1.99 10^3^ mm^2^/s; *D*: 0.89 ± 0.0410^3^ mm^2^/s (32), as D* and *D* values in this cohort were generally below the normal range, suggesting an altered microvascular and microstructural environment. Inflammation appeared to influence these diffusion patterns. In the ischemic hemisphere, D*_i_ was lower in participants with higher levels of inflammation. Although the difference did not reach significance when stratified by *CRP*, it became significant when using *IL-6* levels at high or low inflammation (*p* = 0.031). Similarly, when stratifying by *IL-6*, *D*_*i*_ showed a significant difference between high and low inflammation groups (*p* = 0.047, data not shown). While the perfusion fraction *f* did not significantly differ between groups, it tended to be higher in more inflamed cases. Regarding clinical outcome, diffusion in the contralateral hemisphere (*D*_*c*_) showed a tendency toward higher values in patients with poor functional recovery. Although these values were highly variable, this trend suggests that *D*_*c*_ could be a potential marker of systemic vulnerability or diffuse injury.

**FIGURE 3:**
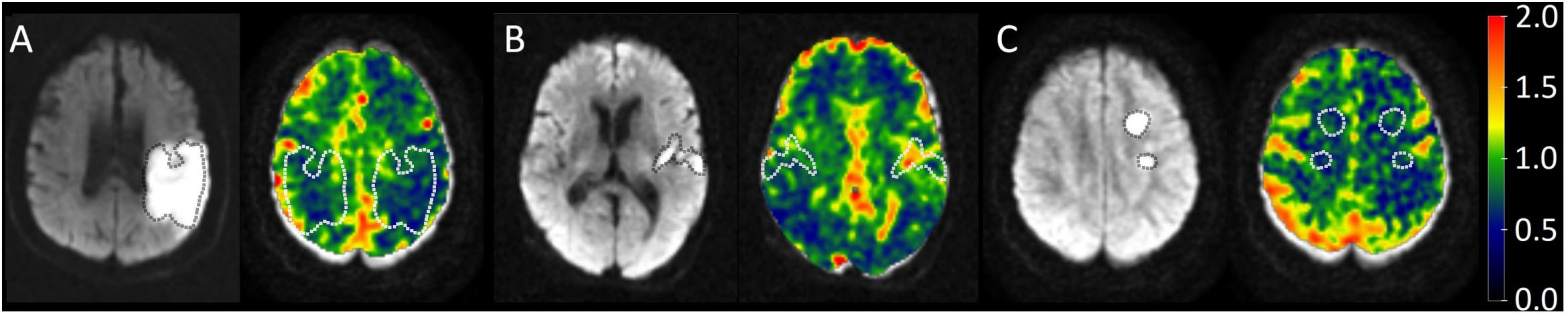
The diffusion-weighted images (DWI) on the left of each panel serve as a guide to identify the ischemic region, which is delineated with a dotted line. On the CVR maps, both the ischemic and contralateral regions are outlined to allow comparison. The color bar indicates lower CVR values in blue and higher values in red. **(A)** Patient 23 demonstrated decreased CVR. **(B)** Patient 11 showed increased CVR. **(C)** Patient 2 showed no significant differences in ROI CVR. Cerebro reactivity maps in three different subjects.

**FIGURE 4:**
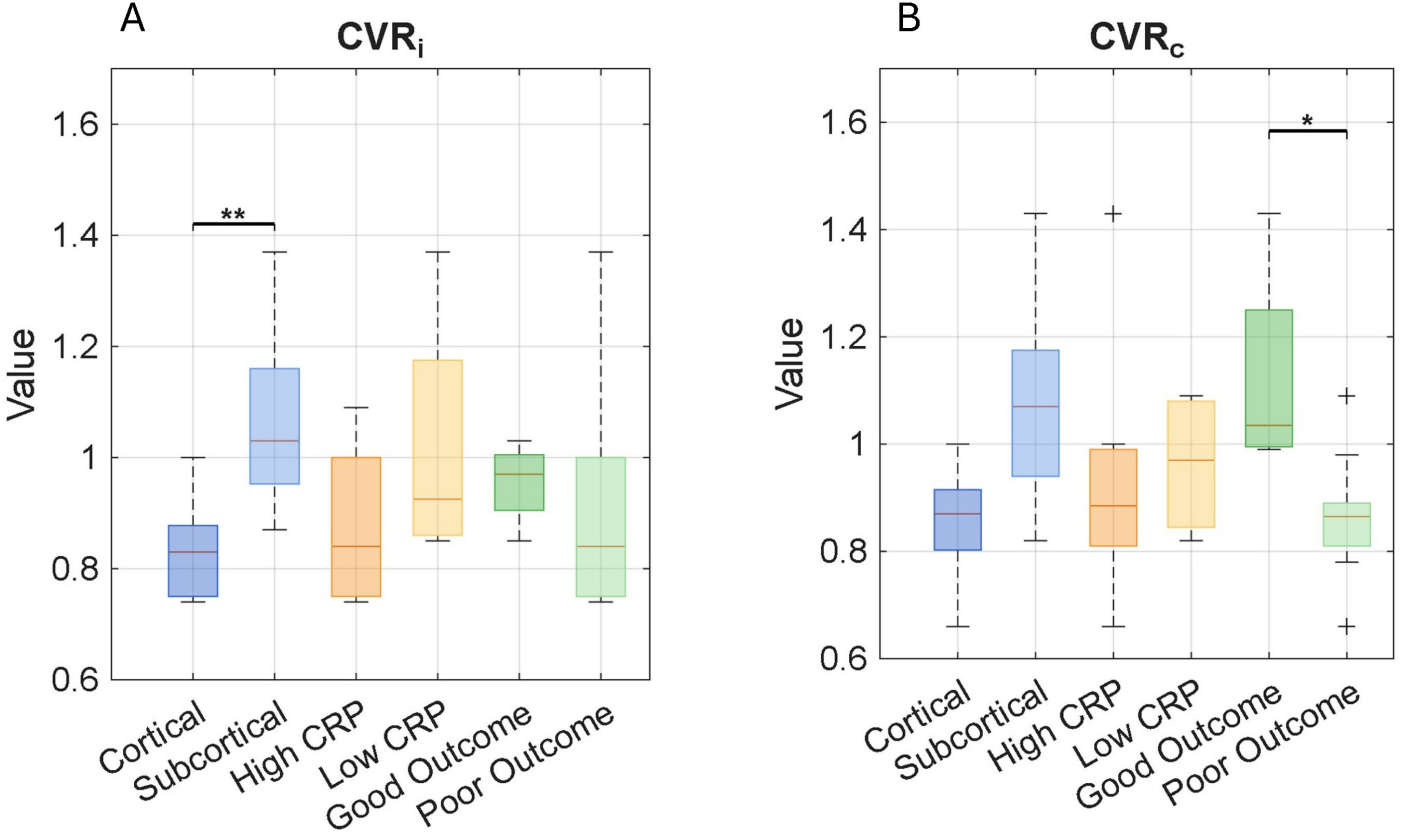
Cerebrovascular reactivity (CVR) estimates derived from resting-state BOLD signal fluctuations are shown across lesion locations (cortical vs. subcortical), levels of systemic inflammation (high vs. low), and functional outcomes (poor vs. good). Each value represents the mean CVR within the entire ischemic ROI and its contralateral counterpart. Boxplots display CVR values in the **(A)** ipsilateral and **(B)** contralateral hemispheres, stratified by each clinical subgroup. Each boxplot shows the median and interquartile range; “**+**” symbols indicate outliers. Asterisks denote statistical significance: *p < 0.05, **p < 0.01. CVR values across lesion location, inflammation, and outcome.

Two patterns were observed in the analysis of differences in ischemic and contralateral CVR values. Some patients displayed decreased reactivity in the contralateral hemisphere compared to the ischemic one, while others exhibited increased values, as illustrated in Figure 3. This possibility has been observed in stenosis patients (26), where some individuals may respond with increased or decreased activity in both severe and mild stenosis, regardless of the condition. This suggests a possible way to understand how the brain reacts to ischemic injury, acting as a measure of dynamic compensatory mechanisms. Figure 4 displays the average CVR when divided by anatomical location, inflammation, and outcome. In the ischemic hemisphere, CVR was notably lower in cortical regions than in subcortical areas (*p* = 0.01). A similar, though not statistically significant, trend appeared in the contralateral hemisphere (*p* = 0.06). Inflammatory status also seemed to affect CVR responses; participants with lower levels of systemic inflammation tended to have higher CVR values in the ischemic hemisphere, indicating a more preserved reactive vascular response under low inflammatory conditions. However, no consistent trend was observed in the contralateral hemisphere with inflammation. Although these findings did not reach statistical significance, they hint at a subtle influence of inflammation on acute vascular reactivity, particularly in ischemic regions. Regarding the clinical outcome, patients with poor functional recovery at 6 months demonstrated significantly higher CVR values in the contralateral hemisphere during the acute phase than those with favorable outcomes (1.12 ± 0.20 vs. 0.85 ± 0.11, *p* = 0.014). These findings emphasize the potential of CVR as a dynamic, non-invasive indicator of cerebrovascular adaptation and contralateral compensation, consistent with previous observations in chronic cerebrovascular conditions (26, 43). In acute stroke, early CVR evaluation could provide prognostic insights into the brain’s ability to adapt to localized ischemia and overall systemic vascular vulnerability.

**FIGURE 5:**
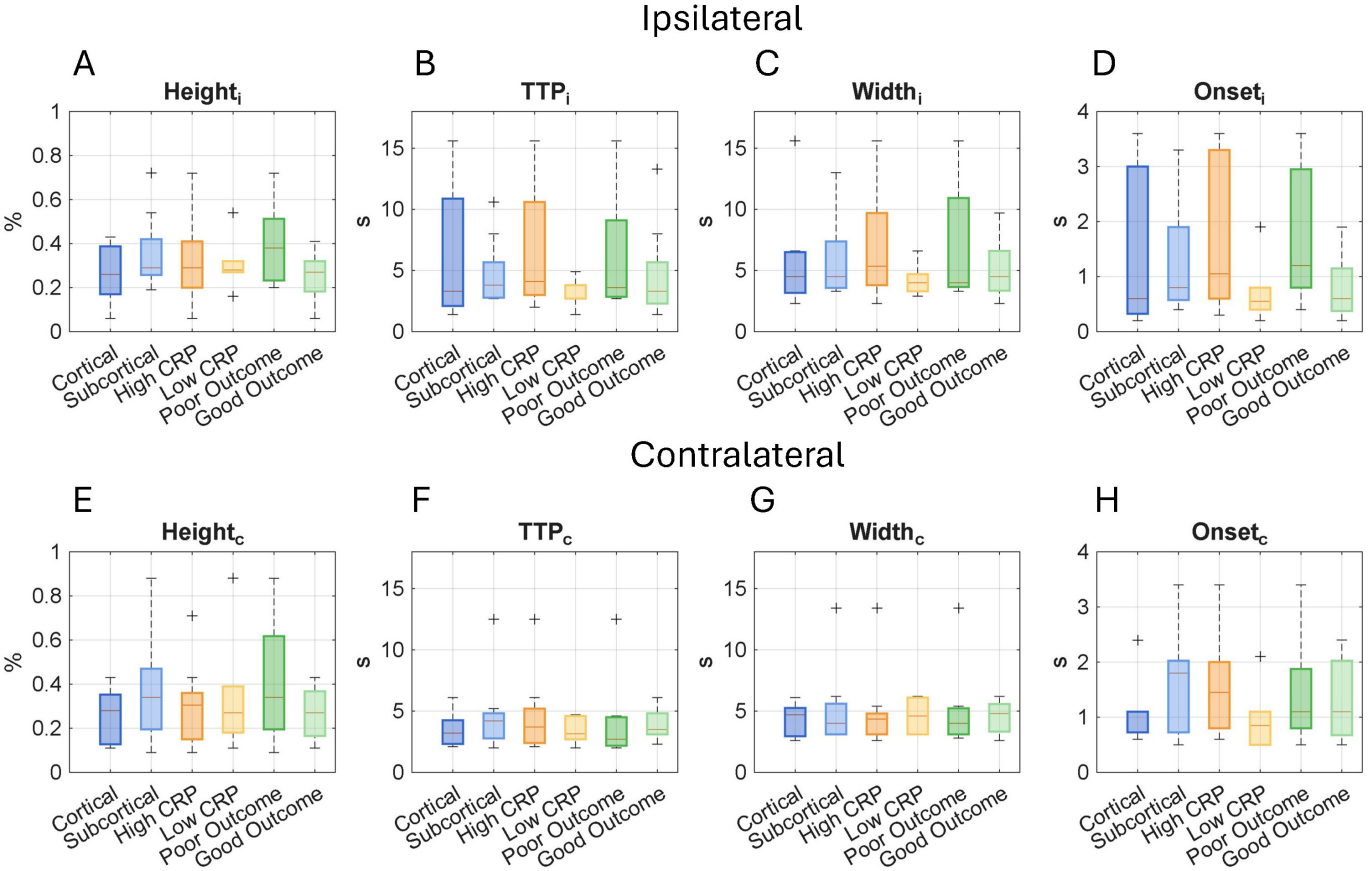
Hemodynamic response function (HRF) metrics across lesion locations (cortical vs. subcortical), systemic inflammation levels (high vs. low), and functional outcomes (good vs. poor). Boxplots display HRF values for the ipsilateral hemisphere (top row) and the contralateral hemisphere (bottom row); The evaluated metrics include **(A,E)** peak amplitude (Height), **(B,F)** time to peak (TTP), **(C,G)** Width, and **(D,H)** Onset time, which together characterize the temporal and amplitude features of the BOLD signal response to the sensorimotor task and reflect neurovascular coupling in the postcentral gyrus of the patients. Each boxplot shows the median and interquartile range, and “**+**” symbols indicate outliers. Comparisons were conducted using the Mann–Whitney U test. No significant differences were reported. HRF imaging measures across lesion location, inflammation, and outcome.

In the non-focal impact, the fMRI neuroimaging results showed that the sensory cortex was activated in 84% of the subjects, demonstrating an activation pattern consistent with expectations for a sensory task at the PCG ROI. Figure 5 presents HRF-derived neurovascular coupling metrics stratified by lesion location, systemic inflammation level, and 6-months functional outcome. When comparing cortical and subcortical cases, height values were consistently higher in subcortical lesions, both in the ischemic and contralateral hemispheres. In relation to systemic inflammation, *TTP*_*i*_ differed significantly: responses were faster in cases with lower inflammation and slower in those with higher inflammatory markers, with statistical significance for *IL-6* (*p* = 0.04) and a trend for *CRP* (*p* = 0.05). Stratification by functional outcome revealed that patients with poor outcomes exhibited a tendency toward increased *Height*, *TTP*, and *Width* on the ischemic side, suggesting a more prolonged or uncoupled hemodynamic response. These patterns suggest that cortical lesions induce localized delays in the neurovascular response, particularly within the ischemic non-focal ROI at the PCG.

**FIGURE 6:**
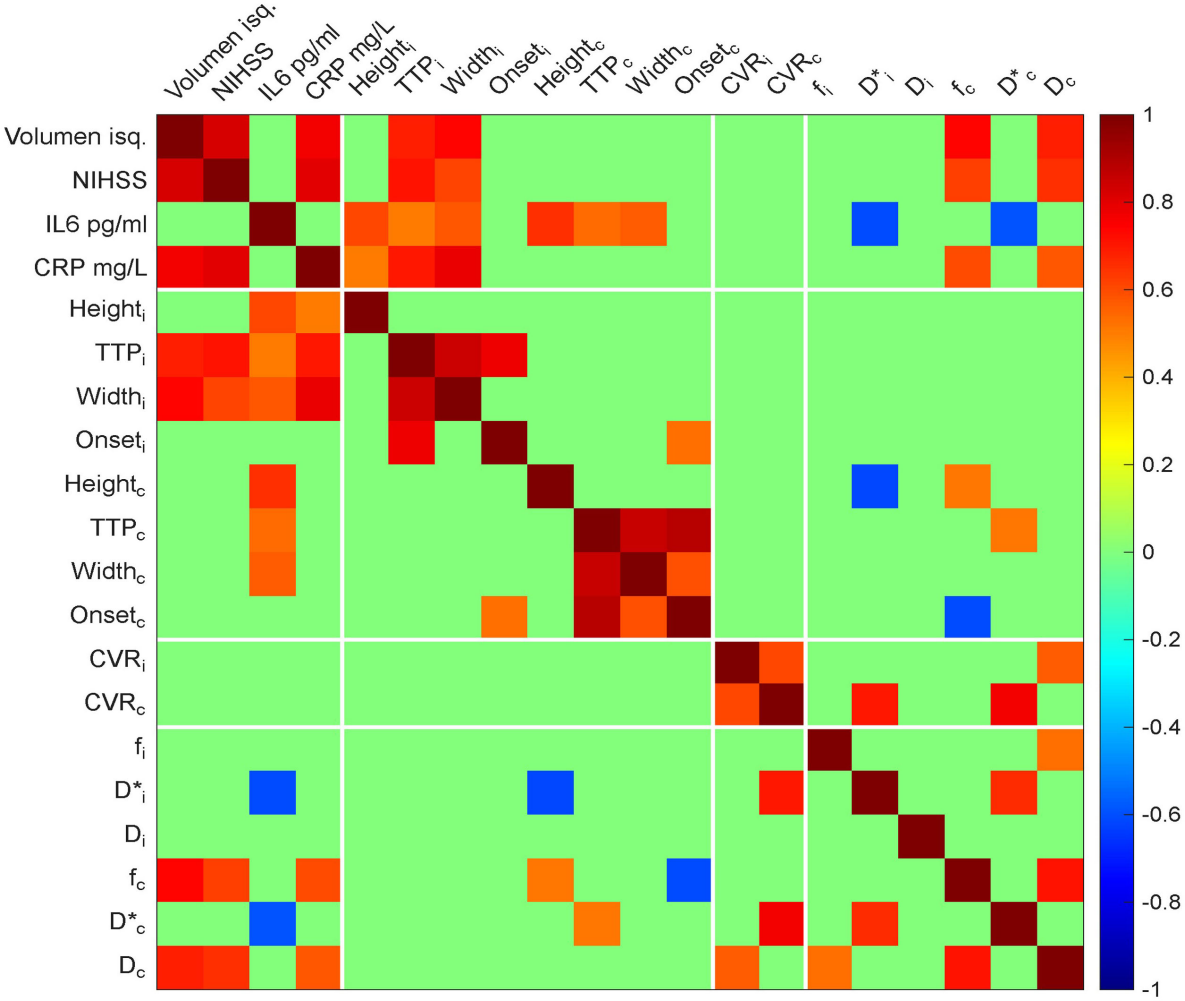
Heat map of pairwise correlations among clinical, biochemical, and neuroimaging variables. Only statistically significant correlations are displayed, defined as those with an absolute Pearson’s correlation coefficient (|r|) > 0.5 and *p* < 0.05. Positive correlations are shown in red and negative correlations in blue, with color intensity reflecting the strength of the association. Clinical variables include NIHSS score and ischemic volume. Biochemical markers include IL-6 and CRP. Neuroimaging parameters are divided into ischemic (i) and contralateral (c) hemispheres and include: height (Height_*i*_, Height_*c*_), time to peak (TTP_*i*_, TTP_*c*_), width (Width_*i*_, Width_*c*_), onset (Onset_*i*_, Onset_*c*_), cerebrovascular reactivity (CVR_*i*_, CVR_*c*_), perfusion fraction (f_*i*_, f_*c*_), pseudodiffussion (D*_i_, D*_c_), and diffusion (D_*i*_, D_*c*_). Correlation heat map between clinical, inflammatory, and neuroimaging in stroke.

In Figure 6, significant associations were observed between clinical severity and inflammatory markers, particularly *CRP* and *IL-6*, as well as HRF metrics, notably *TTP* and *Width* on the ischemic side. Interestingly, *IL-6* was the only inflammatory marker that showed significant correlations with HRF values on the contralateral side. Moreover, *IL-6* was negatively associated with IVIM-derived pseudo diffusion metrics on both the ischemic and contralateral sides. A strong correlation was also found between neuroimaging parameters IVIM D*_c_ and *CVR*_*c*_. Although they are derived from different imaging sequences and aim to measure different physiological aspects, both metrics relate to cerebral blood flow; IVIM D* reflects pseudo-diffusion and *CVR*_*c*_ indicates vascular reactivity, suggesting a convergence of information across imaging methods.

These findings highlight the value of integrating inflammatory and OS biomarkers with advanced hemodynamic and neuroimaging measures to enhance the characterization of the ischemic cascade. This comprehensive approach could provide deeper insights into the systemic effects of stroke through inflammation and neuroimaging, aiding in prognostic approximations.

## 4 Discussion

This study investigated the influence of inflammation and OS on advanced neuroimaging markers and 6-months functional outcomes in patients with cortical or subcortical ischemia. Our findings contribute to filling a critical knowledge gap by offering preliminary insights into the complex interplay between biochemical processes, ischemia location, neuroimaging features, and clinical outcomes. This integrative approach advances our understanding of stroke pathophysiology and may improve prognostic assessment.

Inflammation is a complex immune response triggered by tissue damage. The role of *IL-6* in ischemic stroke pathophysiology is relatively well established (44). Although *IL-6* and *CRP* are functionally linked, and the release of *CRP* depends on the *IL-6* signaling in the liver (9, 45), their associations with stroke parameters may differ. In our study, *CRP* showed a slightly stronger correlation with NIHSS scores and ischemic volume than *IL-6*. This discrepancy may be attributed more to methodological differences with previous works (9, 45) than to underlying biological variability. We assessed *CRP* levels over three consecutive days and used the maximum value to represent peak *CRP* inflammatory response. This approach may have enhanced the observed associations, underscoring the importance of serial biomarker measurements to capture the dynamic nature of post-stroke inflammation better.

Regarding lesion location, our findings suggest that participants with cortical infarcts and heightened inflammatory responses tended to be older and presented with more extensive ischemic damage. In contrast, although inflammatory markers in subcortical regions were lower than those observed in cortical lesions, they remained elevated compared to standard reference values, particularly among patients with unfavorable outcomes. A similar finding was recently reported using a data-driven model to categorize ischemic location, inflammation, and outcome, where cortical regions on both the left and right sides, along with the brainstem, showed higher inflammatory values and poorer outcomes. (46). Differences in *OS*, measured via isoprostanes, between cortical and subcortical infarcts approached statistical significance. Notably, both groups exhibited levels exceeding previously reported reference thresholds for cerebrovascular events, with 459 pg/mL proposed as a potential diagnostic cutoff for cerebrovascular disease (47). This threshold might indicate widespread cellular injury related to cerebrovascular disease and could help distinguish stroke from stroke mimics under challenging cases. To our knowledge, no prior studies have directly compared *OS* levels across cortical and subcortical stroke regions. The trends observed here may be more strongly influenced by lesion volume rather than location alone. Future studies should further investigate how inflammatory and oxidative responses vary according to lesion topography, regional vascular demands, baseline tissue vulnerability, and infarct size.

As illustrated in Figure 2, IVIM imaging revealed location-dependent differences in the perfusion fraction between cortical and subcortical ischemias. Prior studies in healthy individuals have demonstrated that cortical gray matter exhibits higher IVIM perfusion fraction values, while subcortical regions show significantly lower values, approximately half as much (48). These differences may primarily reflect baseline anatomical, functional, and vascular characteristics rather than ischemic mechanisms *per se*. Complementing these findings, increased CVR was observed in subcortical regions, including the brainstem, thalamus, and cerebellum, as depicted in Figure 4. This may represent a region-specific compensatory response to ischemic injury. Previous studies have shown that subcortical structures, particularly those supplied by the posterior circulation, have reduced autoregulatory capacity compared to cortical regions perfused by the anterior circulation (49). While CVR and cerebral autoregulation are distinct mechanisms, both are crucial in maintaining cerebral blood flow (CBF). The increased CVR observed in our cases may indicate a targeted vascular response aimed at maintaining CBF and perfusion in a key area that controls essential and involuntary functions vital for survival, and it needs to be sustained especially under ischemic stress. Nevertheless, the effectiveness of this compensatory response may be limited and could be related to the collateral circulation (26), a point further explored in the context of the outcome. These findings collectively highlight the significance of regional vascular heterogeneity in relation to function and underscore the value of dynamic imaging techniques in evaluating the pathophysiology of ischemic stroke.

Regarding inflammation and neuroimaging parameters, our analysis of nonfocal impact on the postcentral gyrus revealed that HRF parameters were influenced by inflammatory status, as shown in Figure 5. Specifically, higher levels of inflammation were associated with greater stroke severity, as well as prolonged *TTP* and increased *Width* of the HRF curve. These findings suggest that both elevated inflammation and greater lesion burden may contribute to impaired or delayed neurovascular coupling, resulting in broader and slower HRF responses. The HRF represents a dynamic and indirect marker of neurovascular function, capable of capturing disruptions in the neurovascular unit, including interactions between neurons, glial cells, and vasculature, even in regions remote from the primary ischemic site, as observed in this work. To our knowledge, this is the first study to directly assess the combined effect of inflammation and stroke on HRF parameters. Prior work has described resting-state BOLD lags (24, 50) and atypical or non-canonical BOLD responses (23, 51), but these studies did not systematically account for inflammatory markers and employed different imaging modalities and analytic metrics, thereby limiting cross-comparison. We propose that HRF features may serve as useful indirect indicators of neurovascular decoupling, particularly at the cortical level, and could potentially be associated with residual functional capacity depending on the task used. This approach may offer broader applicability to other neurological domains such as cognition and language, supporting early rehabilitation strategies and contributing to prognostic models.

Focal impact explored through IVIM metrics within the ischemic ROI was correlated with both stroke severity and inflammatory markers, as shown in Figure 6. The bilateral reduction in IVIM-derived parameters, especially *D* and D*, when compared to values reported in healthy controls (32, 48), suggests that microvascular alterations in stroke may not be confined to the ischemic core. This observation raises the possibility that such changes may precede the ischemic event, potentially serving as early markers for hypoperfusion or stroke risk stratification. The D*, considered to approximate mean transit time and reflecting the cerebral blood volume to flow ratio (CBV/CBF) (52, 53), was reduced in the ischemic region and showed a significant association with elevated levels of inflammatory markers, particularly *CRP* (Figure 2) and *IL-6* (data not shown). This finding is consistent with previous reports demonstrating decreased D* values in ischemic regions (22). However, other studies have failed to observe significant D* differences between ischemic and non-ischemic tissue in both human and animal models (54, 55). Our findings suggest that D* may be sensitive to inflammation and vascular dysfunction during acute ischemia.

In contrast, the perfusion fraction tended to increase within the ischemic ROI in patients with higher inflammatory levels, including *CRP* and *IL-6*. While previous studies have more commonly reported decreased *f*-values in acute and subacute stroke contexts (22, 54, 55), our observations align with preclinical findings where increased *f* was associated with parenchymal injury from radiation (56), blood–brain barrier (BBB) disruption, and impaired cerebral autoregulation (57, 58). Notably, elevated *f*-values were also observed in the contralateral hemisphere of patients with high levels of *CRP* and *IL-6*. This may reflect a systemic or compensatory perfusion response outside the primary lesion area. Although the precise mechanism remains unclear, this phenomenon could be consistent with global hemodynamic redistribution or compensatory vascular responses (59).

Assessing the contralateral region may provide additional insights into global cerebral stress and compensatory mechanisms activated during the acute phase of stroke. These interactions highlight the potential value of IVIM parameters as complementary markers of stroke pathophysiology, providing a deeper understanding of how inflammation influences microperfusion and how processes such as edema and vascular compensation may mediate tissue responses to ischemia. This is exemplified by the strong correlation observed between D* and *CVR*_*c*_ in Figure 6, suggesting a link between microvascular transit dynamics and cerebrovascular reactivity under inflammatory conditions. Further investigation is warranted to elucidate the acute interplay of these mechanisms and their impact on long-term functional outcomes.

Our report reveals a consistent association between lesion location, inflammation, and advanced neuroimaging metrics. These findings suggest that inflammation may contribute to widespread microvascular dysfunction, reinforcing the need for integrated biomarkers when assessing stroke pathophysiology, especially the inflammation. This sets a foundation for understanding how these mechanisms relate to long-term functional outcomes.

## 5 Conclusion

This case report highlights a critical and often overlooked aspect of ischemic stroke by examining the potential benefits of combining inflammatory markers with early multimodal neuroimaging to enhance understanding of the neuroinflammatory cascade and improve prognostic evaluation. While the contribution of inflammation to stroke pathology has been acknowledged, its specific impact on cerebral hemodynamics and vascular reactivity in the acute phase imaging remains insufficiently explored and characterized. This study provides the first report, to our knowledge, that jointly explores inflammation, oxidative stress, neurovascular coupling, microperfusión, diffusion, and cerebrovascular reactivity in the context of long-term functional outcome. Our observations suggest a novel insight that inflammation influences neuroimaging markers related to neurovascular coupling and pseudodiffusion dynamics, which could be particularly relevant in patients presenting outside standard therapeutic windows. *CVR* and *CRP* measures were directly associated with the outcome at 6 months. However, these preliminary results require validation in larger and more diverse cohorts. These findings support the notion that combining cellular, clinical, and imaging metrics can enhance patient selection, therapeutic management, and prognosis of outcomes. Future studies building on this case series should aim to generate comparable data across different populations, ideally in multicenter settings, to assess whether the proposed integrative metrics of the ischemic process are reproducible and applicable in clinical settings on a large scale.

Combining clinical, biochemical, and advanced neuroimaging data demonstrates the potential of interdisciplinary collaboration to reshape our understanding of stroke effects and prognosis.

## Data Availability

The datasets presented in this article are not readily available because the local committee permits the use of sensitive data exclusively for the original research. Requests to access the datasets should be directed to astrid.cancino@uv.cl.

## Footnotes

1
http://itksnap.org

2
http://dsi-studio.labsolver.org
